# Multi-trait multi-environment Bayesian model reveals G x E interaction for nitrogen use efficiency components in tropical maize

**DOI:** 10.1371/journal.pone.0199492

**Published:** 2018-06-27

**Authors:** Lívia Gomes Torres, Mateus Cupertino Rodrigues, Nathan Lamounier Lima, Tatiane Freitas Horta Trindade, Fabyano Fonseca e Silva, Camila Ferreira Azevedo, Rodrigo Oliveira DeLima

**Affiliations:** 1 Department of Plant Science, Universidade Federal de Viçosa, Viçosa, Minas Gerais, Brazil; 2 Department of Animal Science, Universidade Federal de Viçosa, Viçosa, Minas Gerais, Brazil; 3 Department of Statistics, Universidade Federal de Viçosa, Viçosa, Minas Gerais, Brazil; College of Agricultural Sciences, UNITED STATES

## Abstract

Identifying maize inbred lines that are more efficient in nitrogen (N) use is an important strategy and a necessity in the context of environmental and economic impacts attributed to the excessive N fertilization. N-uptake efficiency (NUpE) and N-utilization efficiency (NUtE) are components of N-use efficiency (NUE). Despite the most maize breeding data have a multi-trait structure, they are often analyzed under a single-trait framework. We aimed to estimate the genetic parameters for NUpE and NUtE in contrasting N levels, in order to identify superior maize inbred lines, and to propose a Bayesian multi-trait multi-environment (MTME) model. Sixty-four tropical maize inbred lines were evaluated in two experiments: at high (HN) and low N (LN) levels. The MTME model was compared to single-trait multi-environment (STME) models. Based on deviance information criteria (DIC), both multi- and single-trait models revealed genotypes x environments (G x E) interaction. In the MTME model, NUpE was found to be weakly heritable with posterior modes of heritability of 0.016 and 0.023 under HN and LN, respectively. NUtE at HN was found to be highly heritable (0.490), whereas under LN condition it was moderately heritable (0.215). We adopted the MTME model, since combined analysis often presents more accurate breeding values than single models. Superior inbred lines for NUpE and NUtE were identified and this information can be used to plan crosses to obtain maize hybrids that have superior nitrogen use efficiency.

## Introduction

Maize is the leading cereal crop in terms of production and, together with rice and wheat, it is one of the most important sources of the global population’s daily caloric requirement [1]. Most of the maize planted in the tropics is grown in areas where soils are acidic and low in nutrients, especially nitrogen (N). Nitrogen plays an essential role in the cycle of most crops and maize requires a large amount of this nutrient. In tropical areas, together with drought, low N conditions represent the major cause of yield loss in maize [2]. On the other hand, some economic and ecological impacts are attributed to excessive N fertilization. Consequently, it is important to improve the N-use efficiency of maize, developing maize cultivars that perform better under low N conditions, to avoid the environmental damage associated with excess N-based fertilizers.

Nitrogen-use efficiency (NUE) is defined as grain yield per unit of N available in the soil and is composed of N-uptake efficiency (NUpE) and N-utilization efficiency (NUtE) [3]. NUpE refers to the quantity of N absorbed by the plant relative to N in the soil, whereas NUtE quantifies the amount of grain produced per unit of N uptake. [4] found that a global average N recovery rate is almost 60%, indicating that two-fifths of N inputs are lost in ecosystems. In a recent review, [5] analyzed the available literature (from a period of over 100 years) on associations between maize yield and plant N dynamics to better understand the plant’s nutrient uptake. They found that fertilizer N accounted for 40% of the total N required by tropical maize. The NUE can be increased through the improvement of NUtE and NUpE. According to [6], at low nitrogen (LN) input, variation in NUtE is more important than variation in N uptake, whereas at high nitrogen (HN) input, the reverse is observed. This highlights the potential of selecting superior maize inbred lines for N use components in order to develop cultivars that are better able to extract the available N in the soil and better exploit that N to produce grain.

The selection of maize inbred lines that are superior for NUE and its components is complex, since they are controlled by many genes and are strongly affected by the environment. Several studies have shown that there is genetic variability for NUE in maize [7, 8, 9] at low and high N levels. However, at low N conditions, there is an increase in the heterogeneity of the fields, generally resulting in higher error variances and lower heritability estimates compared to high N levels [7, 10, 11]. Thus, at low N, the genetic gains are usually lower than at high N. In addition, in experiments with maize under contrasting N levels have shown that there are significant interactions between genotype (G) and environment (N levels; E) for grain yield and NUE components [11, 12, 13, 14]. [10] concluded that maize breeding programs should include LN (low nitrogen level) environments to maximize selection gains when targeting tropical, low N environments. According to [7], the high variance in the G x E interaction emphasizes the need for multi-environment testing to identify N-use efficient cultivars with a broad adaptation to different N levels.

In plant breeding, the genetic evaluation of multiple traits is relevant because superior varieties combine optimal attributes for several traits simultaneously. Despite the fact that data collected in plant breeding studies often present a multi-trait multi-environment structure, these data are rarely analyzed in a combined analysis [15]. Although combined analysis is a better representation of reality, as it takes into consideration the genetic correlations and the G x E interaction, it requires more complex models. In this context, Bayesian inference has become a useful statistical tool to deal with complex models. In the Bayesian approach, the parameters are interpreted as random variables, following the law of probability that reflects a priori knowledge assumption [16]. The Bayesian approach has been successfully used due to the reduced number of biased estimates when there are few observations and furthermore, it produces more precise estimations by intervals [17]. Some studies have shown the potential of the Bayesian approach for genetic evaluation in plant breeding considering multi-trait or multi-environment models [18, 19, 20, 21]. However, there are few studies combining multi-trait models under a multi environment under a Bayesian framework. Additionally, to the best of our knowledge, there is no study combining NUE-components in multiple environments in the same model in maize. In the literature, generally there are investigations with single traits (especially grain yield) in multi environments or multi traits in a single environment.

Toward this orientation, we aimed to propose a multi-trait multi-environment Bayesian model to estimate genetic parameters for N-uptake efficiency and N-utilization efficiency under contrasting N levels in the soil as well as to perform comparisons with single trait models. We aimed also to identify superior maize inbred lines for N-uptake and N-utilization efficiencies.

## Materials and methods

### Plant material

The genetic material consisted of 64 tropical maize inbred lines. These maize inbred lines represent a set of diverse germplasm from the maize breeding program of Universidade Federal de Viçosa (UFV), Viçosa, Minas Gerais state, Brazil. They were obtained from different sources of tropical maize germplasm: commercial tropical maize hybrids, maize populations and maize open-pollinated varieties (OPV; Table 1). It is important to mention that, in Brazil, it is allowed to use any commercial variety as germplasm source for breeding purposes and the development of new maize inbred lines; therefore it is possible to use maize hybrids, except the ones with transgenic events (10^th^ article, of the third paragraph of the No. 9456 Brazilian Law of April 25^th^, 1997). The genetic sources used for our program to develop new inbred lines are conventional varieties. The 64 maize inbred lines were developed using a modified pedigree method in the same region where they were evaluated. Most of tropical hybrids used as source to develop the inbred lines studied in this work are recommend to tropical areas; consequently, they are adapted to the region where the inbred lines were studied. The CMS28 is a tropical maize population introduced in Brazil from International Maize and Wheat Improvement Center (CIMMYT) by the Brazilian Agricultural Research Corporation (EMBRAPA), Maize and Sorghum unit, in the 70’s [22]. The Nitroflint, CMS50 and BR106 are tropical maize populations developed by EMBRAPA Maize and Sorghum. The Nitroflint population is adapted to low N soil and, it has been used as source germplasm for the development of new inbred lines to tolerance to low N in soil [23]. The CMS50 is a tropical maize population produced by the intercrossing of three single-cross and two double-crosses [24]. In addition, the BR106 is a tropical open-pollinated variety produced by the intercrossing of three Brazilian varieties (Maya, Centralmex and Compose Dent) and an exotic introduction (Tuxpeño 1) [25]. It is cultivated by some small farmers in Brazil and used in some recurrent selection programs in public sector.

**Table 1:** Name, endosperm type, pedigree/origin and source type of the 64 tropical maize inbred lines.

Entry number	Inbred line	Endosperm type	Pedigree/Origin	Source type
1	VML001	Flint	GM001	Tropical Hybrid
2	VML002	Flint	GM004	Tropical Hybrid
3	VML003	Flint	GM013	Tropical Hybrid
4	VML004	Flint	GM012	Tropical Hybrid
5	VML005	Semi-flint	GM029	Tropical Hybrid
6	VML006	Flint	GM007	Tropical Hybrid
7	VML007	Semi-flint	GM008	Tropical Hybrid
8	VML008	Flint	GM027	Tropical Hybrid
9	VML009	Dent	GM020	Tropical Hybrid
10	VML010	Dent	GM033	Tropical Hybrid
11	VML012	Flint	BR106	OPV
12	VML013	Semi-flint	GM029	Tropical Hybrid
13	VML014	Flint	GM029	Tropical Hybrid
14	VML015	Flint	GM032	Tropical Hybrid
15	VML016	Flint	GM022	Tropical Hybrid
16	VML017	Flint	BR106	OPV
17	VML018	Dent	GM017	Tropical Hybrid
18	VML019	Flint	GM008	Tropical Hybrid
19	VML020	Flint	CMS28	Tropical Population
20	VML021	Flint	GM034	Tropical Hybrid
21	VML022	Semi-flint	GM035	Tropical Hybrid
22	VML023	Flint	GM028	Tropical Hybrid
23	VML024	Flint	CMS50	Tropical Population
24	VML025	Flint	GM018	Tropical Hybrid
25	VML026	Flint	GM028	Tropical Hybrid
26	VML027	Flint	GM036	Tropical Hybrid
27	VML028	Flint	GM007	Tropical Hybrid
28	VML029	Semi-flint	GM027	Tropical Hybrid
29	VML032	Flint	GM018	Tropical Hybrid
30	VML033	SemiDent	GM011	Tropical Hybrid
31	VML034	Flint	GM010	Tropical Hybrid
32	VML035	Flint	GM026	Tropical Hybrid
33	VML036	Flint	GM005	Tropical Hybrid
34	VML037	Flint	GM016	Tropical Hybrid
35	VML040	Flint	GM014	Tropical Hybrid
36	VML043	Flint	GM012	Tropical Hybrid
37	VML045	Semi-flint	GM002	Tropical Hybrid
38	VML048	Flint	Nitroflint	Tropical Population
39	VML050	Flint	GM035	Tropical Hybrid
40	VML051	Semi-dent	GM022	Tropical Hybrid
41	VML052	SemiFlint	GM019	Tropical Hybrid
42	VML054	Flint	GM034	Tropical Hybrid
43	VML055	Dent	GM027	Tropical Hybrid
44	VML076	Flint	GM031	Tropical Hybrid
45	VML077	Semi-flint	GM030	Tropical Hybrid
46	VML081	Flint	GM003	Tropical Hybrid
47	VML084	Dent	GM001	Tropical Hybrid
48	VML086	Flint	GM015	Tropical Hybrid
49	VML110	Flint	GM024	Tropical Hybrid
50	VML125	Flint	GM009	Tropical Hybrid
51	VML182	Flint	GM006	Tropical Hybrid
52	VML188	Flint	CMS50	Tropical Population
53	L013	Flint	GM023	Tropical Hybrid
54	L015	Dent	GM012	Tropical Hybrid
55	L034	Flint	GM027	Tropical Hybrid
56	L037	Flint	CMS50	Tropical Population
57	L038	Flint	CMS28	Tropical Population
58	L040	Flint	CMS28	Tropical Population
59	L045	Flint	GM007	Tropical Hybrid
60	L052	Flint	GM016	Tropical Hybrid
61	L054	Flint	GM028	Tropical Hybrid
62	L059	Semi-flint	GM016	Tropical Hybrid
63	L061	Flint	GM025	Tropical Hybrid
64	L062	Flint	GM021	Tropical Hybrid

### Field experiments

The 64 tropical maize inbred lines were evaluated in 2014 at UFV Experimental Station in Coimbra (latitude 20°51'24''S; longitude 42°48'10''W; altitude of 720 m asl), located in southwest Minas Gerais state, Brazil. Maize inbred lines were evaluated in two independent experiments under contrasting levels of N: low nitrogen (LN) and high nitrogen (HN). In this context, there is a totally different environmental condition for plants, thus characterizing different environments, mainly because they are very contrasting nitrogen levels.

The design of each experiment was an 8 x 8 lattice square with two replications and two-row plots. The plot size was 6.4 m^2^ (4 m long with 0.8 m row spacing and 0.2 m plant spacing). At the LN level, 30 kg ha^-1^ of N was applied. At the HN level, 180 kg ha^-1^ was applied. Trait management was the same for all N level experiments, employing standard agricultural practices.

Grain yield was recorded from all ears on the plot at physiological maturity. Ears were shelled, the grain weight and grain moisture percentage were recorded, and grain yield (kg ha^-1^) was calculated at 145 g kg^-1^ moisture. At physiological maturity, five representative plants were harvested from each plot by cutting them close to the soil surface. All plant stover together with cobs (with kernels removed) at maturity were chopped and oven-dried to a constant weight at 70°C for 72 hours. The harvest ears were also oven-dried at 70°C for 72 hours. Grain and stover samples were milled using an analytical mill and analyzed for N according to the Kjeldahl method [26].

The components of NUE were calculated according to [3]: N-uptake efficiency was calculated as the ratio of the total N (kg ha^-1^) in the aboveground biomass to total N in the soil (kg ha^-1^) and N-utilization efficiency was calculated as the ratio of grain yield (kg ha^-1^) to total N (kg ha^-1^) in the aboveground biomass.

### Statistical analysis

N-uptake efficiency and N-utilization efficiency were analyzed using single and multi-trait models via the Markov Chain Monte Carlo (MCMC) Bayesian approach. The idea was to compare: (i) the full model (considering the interaction between the genotypes and N levels) with the null model (not considering the interaction); (ii) the estimates of the genetic parameters from single- and multi-trait models at LN and HN levels.

The multi-trait multi-environment model was given by:

$$y=X\beta +Z_{1}r+Z_{2}b+Z_{3}u+\varepsilon$$ (1)

Which can be rewritten as:

$$\left(\begin{array}{c} Y_{1}\\ \cdots \\ Y_{2} \end{array}\right)=X\left(\begin{array}{c} \beta (l,1)\\ \beta (h,1)\\ \cdots \cdots \\ \beta (l,2)\\ \beta (h,2) \end{array}\right)+Z_{1}\left(\begin{array}{c} r_{1}(l,1)\\ r_{2}(h,1)\\ \cdots \cdots \\ r_{1}(l,2)\\ r_{2}(h,2) \end{array}\right)+Z_{2}\left(\begin{array}{c} b_{11}(l,1)\\ \vdots \\ b_{82}(h,1)\\ \cdots \cdots \\ b_{11}(l,2)\\ \vdots \\ b_{82}(h,2) \end{array}\right)+Z_{3}\left(\begin{array}{c} u(l,1)\\ u(h,1)\\ \cdots \cdots \\ u(l,2)\\ u(h,2) \end{array}\right)+\left(\begin{array}{c} \varepsilon (l,1)\\ \varepsilon (h,1)\\ \cdots \cdots \\ \varepsilon (l,2)\\ \varepsilon (h,2) \end{array}\right)$$ (2)

Where: l represents low and *h* represents high N; 1 represents NUpE and 2 represents NUtE; y is the vector of the phenotypic values of the traits (NUpE and NUtE); β is the vector of systematic effects of environment (N levels), **β** ~ N(**μ**, **I** ⊗ ∑_β_); r is the vector of the replication inside the environment effect, **r** ~ N(**0**, **I** ⊗ ∑_r_); b is the vector of random effects of block inside replication inside environment, **b** ~ N(**0**, **I** ⊗ ∑_*b*_); u is the vector of random effects of genotypes, **u** ~ N(**0**, **I** ⊗ ∑_u_); ɛ is the vector of random residual effects, **ε** ~ N(**0**, **I** ⊗ ∑_*ε*_). X is the incidence matrix of environment (N level) effects, Z_1_ is the incidence matrix of replication inside the environment effect, Z_2_ is the incidence matrix of block inside replication inside environment effects and Z_3_ is the incidence matrix of genotype effects.

The (co)variance matrix estimates are given by:

$$\sum_{u}=\left(\begin{array}{ccccc} \sigma_{ul(1)}^{2} & \sigma_{ul,h(1)} & \ldots & \sigma_{ul(1,2)} & \sigma_{ul,h(1,2)}\\ \sigma_{ul,h(1)} & \sigma_{uh(1)}^{2} & \ldots & \sigma_{ul,h(1,2)} & \sigma_{uh(1,2)}\\ \vdots & \vdots & \ddots & \vdots & \vdots \\ \sigma_{ul(1,2)} & \sigma_{ul,h(1,2)} & \ldots & \sigma_{ul(2)}^{2} & \sigma_{ul,h(2)}\\ \sigma_{ul,h(1,2)} & \sigma_{uh(1,2)} & \ldots & \sigma_{ul,h(2)} & \sigma_{uh(2)}^{2} \end{array}\right)$$

$$\sum_{r}=\left(\begin{array}{ccccc} \sigma_{rl(1)}^{2} & \sigma_{rl,h(1)} & \ldots & \sigma_{rl(1,2)} & \sigma_{rl,h(1,2)}\\ \sigma_{rl,h(1)} & \sigma_{rh(1)}^{2} & \ldots & \sigma_{rl,h(1,2)} & \sigma_{rh(1,2)}\\ \vdots & \vdots & \ddots & \vdots & \vdots \\ \sigma_{rl(1,2)} & \sigma_{rl,h(1,2)} & \ldots & \sigma_{rl(2)}^{2} & \sigma_{rl,h(2)}\\ \sigma_{rl,h(1,2)} & \sigma_{rh(1,2)} & \ldots & \sigma_{rl,h(2)} & \sigma_{rh(2)}^{2} \end{array}\right)$$

$$\sum_{b}=\left(\begin{array}{ccccc} \sigma_{bl(1)}^{2} & \sigma_{bl,h(1)} & \ldots & \sigma_{bl(1,2)} & \sigma_{bl,h(1,2)}\\ \sigma_{bl,h(1)} & \sigma_{bh(1)}^{2} & \ldots & \sigma_{bl,h(1,2)} & \sigma_{bh(1,2)}\\ \vdots & \vdots & \ddots & \vdots & \vdots \\ \sigma_{bl(1,2)} & \sigma_{bl,h(1,2)} & \ldots & \sigma_{bl(2)}^{2} & \sigma_{bl,h(2)}\\ \sigma_{bl,h(1,2)} & \sigma_{bh(1,2)} & \ldots & \sigma_{bl,h(2)} & \sigma_{bh(2)}^{2} \end{array}\right)$$

$$\sum_{\varepsilon }=\left(\begin{array}{ccccc} \sigma_{\varepsilon l(1)}^{2} & \sigma_{\varepsilon l,h(1)} & \ldots & \sigma_{\varepsilon l(1,2)} & \sigma_{\varepsilon l,h(1,2)}\\ \sigma_{\varepsilon l,h(1)} & \sigma_{\varepsilon h(1)}^{2} & \ldots & \sigma_{\varepsilon l,h(1,2)} & \sigma_{\varepsilon h(1,2)}\\ \vdots & \vdots & \ddots & \vdots & \vdots \\ \sigma_{\varepsilon l(1,2)} & \sigma_{\varepsilon l,h(1,2)} & \ldots & \sigma_{\varepsilon l(2)}^{2} & \sigma_{\varepsilon l,h(2)}\\ \sigma_{\varepsilon l,h(1,2)} & \sigma_{\varepsilon h(1,2)} & \ldots & \sigma_{\varepsilon l,h(2)} & \sigma_{\varepsilon h(2)}^{2} \end{array}\right)$$

where: l represents LN and *h* represents HN; 1 represents NUpE and 2 represents NUtE. We assumed that the variance-covariance matrices follow an inverted Wishart distribution, which was used as a priori to model the variance-covariance matrix [27].

The model was implemented in the “MCMCglmm” R package [28, 29] of R software (R Development Core Team). A total of 1,000,000 samples were generated, and assuming a burn-in period and sampling interval of 500,000 and 5 iterations, respectively, this resulted in a final total of 100,000 samples. The convergence of MCMC was checked by the criterion of [30], which was performed using the “boa” [31] and “CODA” (convergence diagnosis and output analysis) [32] R packages. Even though Bayesian and frequentist frameworks are not directly compared, mainly in the field of Genetics and Breeding [17], the same models were also fitted based on REML (Restricted Maximum Likelihood) estimation method. However, the convergence was not achieved through AI (Average Information) and EM (Expectation-Maximization) algorithms.

The full (considering the inbred lines x N levels interaction) models were compared with the null (not considering the interaction) models by the deviance information criterion (DIC) proposed by [33]: $\mathrm{DIC}=D(\bar{\theta )}+2p_{D}$, where $D(\bar{\theta )}$ is a point estimate of the deviance obtained by replacing the parameters with their posterior means estimates in the likelihood function and *p*_*D*_ is the effective number of parameters in the model. Models with smaller DIC should be preferred to models with higher DIC.

Variance components, broad-sense heritability *per plot*, coefficients of residual and genetic variation, the variation index and the genotypic correlation coefficients between traits and breeding values were calculated from the posterior distribution. The package “boa” [31] of R software was used to calculate the highest posterior density (HPD) intervals for all parameters. Posterior estimates for the broad-sense heritability of NUpE and NUtE for each interaction were calculated from the posterior samples of variance components obtained by the model, using the expression:

$$h^{2(i)}=\frac{\sigma_{g}^{2(i)}}{\left(\sigma_{g}^{2(i)}+\sigma_{r}^{2(i)}+\sigma_{b}^{2(i)}+\sigma_{\varepsilon }^{2(i)}\right)},$$

Where: $\sigma_{g}^{2(i)}$, $\sigma_{r}^{2(i)}$, $\sigma_{b}^{2(i)}$ and $\sigma_{\varepsilon }^{2(i)}$ are the genetic, replication, block inside replication and residual variances for each iteration, respectively. The genetic association between the pairs of traits in each environment was calculated as:

$$r_{l(1,2)}=\frac{\sigma_{gl(1,2)}}{\sqrt{\sigma_{gl(1)}^{2}\sigma_{gl(2)}^{2}}}\;\left(\mathrm{genetic}\;\mathrm{correlation}\;\mathrm{between}\;\mathrm{traits}\;\mathrm{at}\;\mathrm{LN}\;\mathrm{level}\right)$$

$$\mathrm{and}\;r_{h(1,2)}=\frac{\sigma_{gh(1,2)}}{\sqrt{\sigma_{gh(1)}^{2}\sigma_{gh(2)}^{2}}}\;(\mathrm{genetic}\;\mathrm{correlation}\;\mathrm{between}\;\mathrm{traits}\;\mathrm{at}\;\mathrm{HN}\;\mathrm{level}).$$

## Results and discussion

All chains achieved convergence via the criterion of [30]. According to the deviance information criteria (DIC), there was positive evidence of interactions between inbred lines and N levels for all models analyzed (Table 2). The inbred lines x N levels interaction for plant traits indicates that the best genotypes under LN are not the same as at an HN level [34]. Thus, when selecting maize inbred lines, the target environment must be considered.

**Table 2:** Deviance information criteria for the full (considering G x E interaction) and null (not considering the interaction) models.

Model	Trait^a^	Deviance information criteria (DIC)	
		Full model	Null model
Multi-trait	NUpE, NUtE	604.79	1958.81
Single-trait	NUpE	102.42	197.35
Single-trait	NUtE	226.89	1814.98

^a^NUpE (N-uptake efficiency, kg ha^-1^ of N absorbed/kg ha^-1^ of N supply); NUtE (N-utilization efficiency, kg ha^-1^ of grain/kg ha^-1^ of N in the plant at maturity).

For the full model, the average values for NUpE were 0.499 and 2.103, and for NUtE were 28.842 and 30.318, under HN and LN, respectively (Table 3). In the multi-trait multi-environment (MTME) model, NUpE at HN and LN were found to be weakly heritable with Bayesian credible intervals (probability of 95%): h^2^ = 4.177x10^-5^ to 0.090 and h^2^ = 6.644x10^-5^ to 0.135, posterior modes: h^2^ = 0.016 and h^2^ = 0.023, at HN and LN, respectively. Just a comment about this, we would like to emphasize that the low heritability observed only for NUpE did not depend on the number of evaluated samples, since the used Bayesian framework is essentially recommended for situations involving small sample sizes. The NUtE at HN was found to be highly heritable whereas at LN it was moderately heritable, with credible intervals (probability of 95%) ranging from: h^2^ = 0.240–0.674 and h^2^ = 0.084–0.396, posterior modes: h^2^ = 0.490 and h^2^ = 0.215, respectively. The difference between mean, mode and median heritability estimates (Tables 3 and 4) reflects some lack of symmetry in the posterior distribution estimates [21]. In our study, we found higher heritability for NUpE at LN and higher heritability for NUtE at HN level. In a study on maize hybrids in three N levels, [7] also found that, for NUpE, the h^2^ increased with a decrease in N level, whereas for NUtE, the highest heritability was estimated at an HN level.

**Table 3:** Posterior inferences for the mean and genetic variance; the mode, mean, median and higher posterior density (HPD) interval of the broad-sense heritability; and the mode, mean, median and higher posterior density (HPD) interval of the genetic correlation, considering the proposed multi-trait multi-environment model.

Trait^a^	N level	Mean	$\sigma_{g}^{2}$	h^2^2			HPD (95%)	
				Mode	Mean	Median	Lower	Upper
NUpE	HN	0.499	0.095	0.016	0.038	0.032	4.177E-05	0.090
NUpE	LN	2.103	0.183	0.023	0.057	0.049	6.644E-05	0.135
NUtE	HN	28.842	40.323	0.490	0.463	0.475	0.240	0.674
NUtE	LN	30.318	27.534	0.215	0.233	0.229	0.084	0.396
				Genotypic correlation			HPD (95%)	
				Mode	Mean	Median	Lower	Upper
NUpExNUtE	HN	-	-	-0.052	-0.051	-0.052	-0.330	0.241
NUpExNUtE	LN	-	-	0.018	-0.002	-0.003	-0.328	0.338

^a^NUpE (N-uptake efficiency, kg ha^-1^ of N absorbed/kg ha^-1^ of N supply); NUtE (N-utilization efficiency, kg ha^-1^ of grain/kg ha^-1^ of N in the plant at maturity).

**Table 4:** Posterior inferences for the mean and genetic variance; the mode, mean, median and higher posterior density (HPD) interval of the broad-sense heritability, considering the single-trait multi-environment models.

Trait^a^	N level	Mean	$\sigma_{g}^{2}$	h^2^			HPD (95%)	
				Mode	Mean	Median	Lower	Upper
NUpE	HN	0.492	0.051	0.014	0.039	0.033	1.227E-05	0.095
NUpE	LN	2.111	0.156	0.067	0.092	0.084	2.064E-04	0.199
NUtE	HN	28.872	40.854	0.500	0.462	0.476	0.223	0.673
NUtE	LN	30.336	29.320	0.226	0.239	0.235	0.081	0.411

^a^NUpE (N-uptake efficiency, kg ha^-1^ of N absorbed/kg ha^-1^ of N supply); NUtE (N-utilization efficiency, kg ha^-1^ of grain/kg ha^-1^ of N in the plant at maturity).

In the current study, the posterior mean of the genetic correlation between NUpE and NUtE was not significantly different from zero (95% credible intervals) under both N levels (HN: -0.330 to 0.241; LN: -0.328 to 0.338) (Table 3). Although some studies consider NUpE and NUtE to be not independent from a statistical point of view, in the present study the opposite was observed. [7] evaluated N-utilization efficiency and N-uptake efficiency in two sets of maize hybrids, one produced from crosses among maize inbred lines selected at HN and another set of hybrids from inbred lines selected at LN. The authors found that at LN, for hybrids from lines selected at HN, N-utilization efficiency was positively related to NUpE, whereas at HN, it was negatively and highly related to NUpE. However, for hybrids from lines selected at LN, there was no correlation among components of NUE and the components of NUE were independent. [12] also found that the genetic correlation between components of NUE was not significant and each was approximately equally related to NUE. Results depend on the type (and set) of genotypes (populations, hybrids, recombinant inbred lines, inbred lines) evaluated. [35], in a review of the genetics of NUE in crop plants, stated that maize studies can be divided into two groups, the comparison studies of hybrids from different eras and the ones related to inbred lines use or mapping populations. The authors also discussed that remains difficult to determine whether maize hybrids respond differently than inbred lines to fertilizer N. According to [6], if only elite hybrids are evaluated, probably specific genes for adaptation to low nitrogen condition have been lost due to selection in favorable conditions over the years. Thus, according to the results from the present study, for maize inbred lines NUpE cannot be used for indirect selection of NUtE, and vice versa, and the genetic improvement for NUE in maize breeding programs should consider the improvement of both NUE-components, NUtE and NUpE.

For the single- and multi-trait models, the means estimates for NUpE and NUtE were always higher in LN inputs than in HN (Tables 3 and 4), with a more pronounced difference between environments for NUpE than NUtE. [6] reported that NUE is higher at LN level than at HN level and this is due to the fact that the efficiency of N decreases as the fertilization level increases. These authors also mentioned that *quantitative trait loci* (QTL) studies tend to confirm that variation in NUtE is greater than variation in NUpE at LN environments and the opposite occurs at HN environments. In the present study, the genetic variance was greater for NUpE in LN and greater for NUtE in HN (Tables 3 and 4). These different values between the environments (N levels) may be due to the biological response of the inbred lines to the nutritional N stress. [36] verified that the genes responsible for the control of NUE are expressed according to the availability of the nutrient to the plant and consequently, the magnitude of genetic variability is expressed differently in contrasting environments. It was noticed here that posterior inferences for the genetic parameters obtained through the multi-trait model were very similar to the ones obtained through the single-trait models (Tables 3 and 4), this could be due to the lack of correlation between traits.

**Fig 1:**
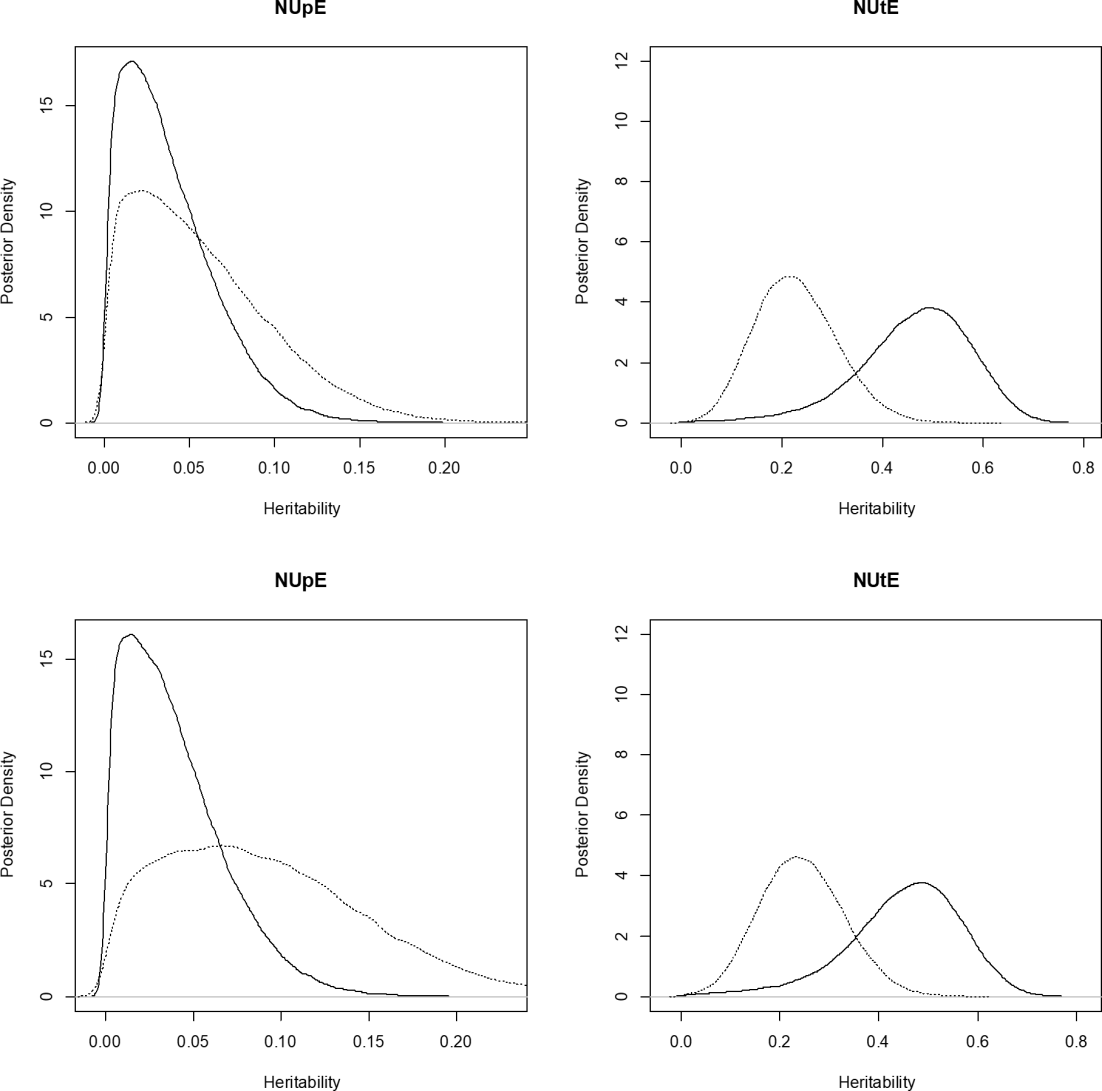
**Posterior density for the multi-trait multi-environment model above (left: NUpE and right: NUtE) and for each of the single-trait multi-environment models below (left: NUpE and right: NUtE)**. The solid line represents the posterior density for the HN level, while the dotted line represents the posterior density for the LN level.

**Fig 2:**
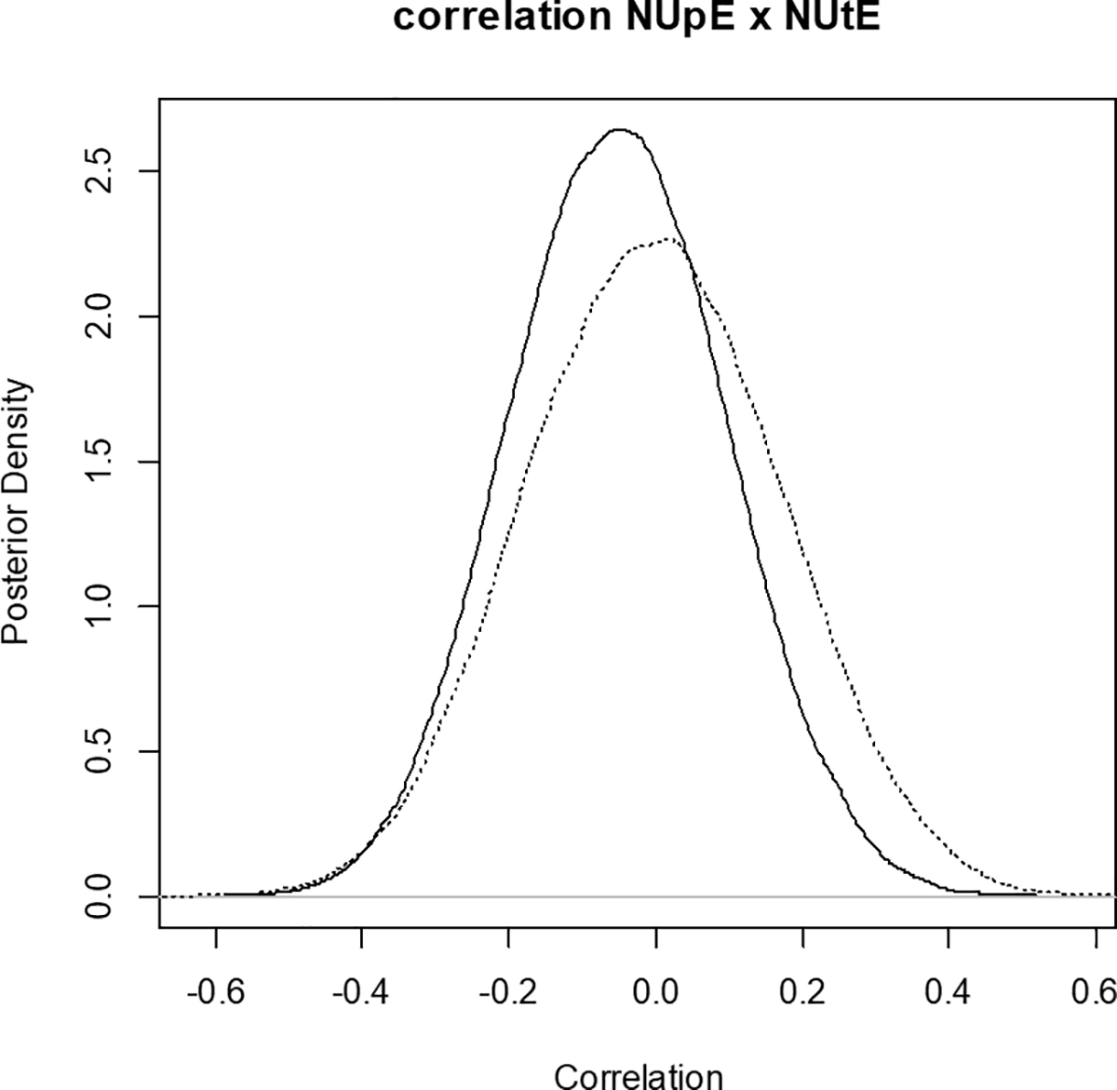
Posterior density for the genotypic correlation between the traits nitrogen-uptake efficiency (NUpE) and nitrogen-utilization efficiency (NUtE) for the multi-trait multi-environment model. The solid line represents the posterior density for the HN level, while the dotted line represents the posterior density for the LN level.

One of the advantages of Bayesian methods is the ability to access the posterior density intervals of genetic parameters (Figs 1 and 2). The breeding values of the maize inbred lines for each trait and their highest posterior density (HPD) intervals were obtained and were used to assist in the selection of genotypes.

The relative variation index is the ratio of the coefficient of genotypic variation to the coefficient of residual variation (CVg/CVe). Relative variation indices that are greater than the unit suggest that genetic variation is more influential than residual variation. This was observed in this study for both traits but only in HN input, suggesting that there was a greater influence of the residual variation under low N level than high N level. Highest CVe and CVg were observed for NUpE in HN input, whereas lowest CVg was observed for NUtE in LN input (Table 5).

**Table 5:** Coefficient of residual variation (CVe, %), coefficient of genotypic variation (CVg, %) and relative variation index (CVg/CVe) for the multi-trait multi-environment model and the single-trait multi-environment models.

Model	Trait^a^	N level	CVe (%)	CVg (%)	CVg/CVe
Multi-trait	NUpE	HN	51.36	61.66	1.20
	NUpE	LN	21.61	20.35	0.94
	NUtE	HN	19.53	22.02	1.13
	NUtE	LN	29.49	17.31	0.59
Single-trait	NUpE	HN	38.31	45.67	1.19
	NUpE	LN	19.18	18.74	0.98
	NUtE	HN	19.57	22.14	1.13
	NUtE	LN	29.33	17.85	0.61

^a^NUpE (N-uptake efficiency, kg ha^-1^ of N absorbed/kg ha^-1^ of N supply); NUtE (N-utilization efficiency, kg ha^-1^ of grain/kg ha^-1^ of N in the plant at maturity).

We have chosen to adopt the MTME model for the inbred lines selection since a combined analysis takes more data into consideration and consequently is often more accurate to predict breeding values than single models [37, 38]. According to [39], the increase in accuracy with the use of multi-trait best linear unbiased prediction (BLUP) analysis compared with single-trait analysis is proportional to the difference between the genetic and environmental correlations of the analyzed traits. In order to compare the ranking of the most efficient tropical maize inbred lines between multi- and single-trait models for each trait in a given environment, the percentage of coincidence of the top 10 inbred lines list (not considering their order in the list) was calculated. For NUpE the coincidence percentage between models was 80% and 70% at LN and HN, respectively. The coincidence percentage for NUtE was 100% under both N levels. The high percentage of coincidence between rankings of the multi- and single-trait models may be, as already mentioned, due to the fact that the genetic correlation between the traits was not significant. Genetic merit estimates of individuals, whether or not they consider genomic information, are in general estimated more accurately when they are based on analyses that consider all the traits simultaneously–that is, under a multivariate approach [40, 41]. In a study with popcorn half-sib families, [42] found that multi-trait BLUP (Best Linear Unbiased Prediction) was more accurate and efficient in family selection than single-trait BLUP. The effect of the G x E interaction was significant, which indicates that the maize inbred lines responded differently to N levels. In order to compare the ranking of inbred lines between the environments (HN and LN) for each trait in a given model, the percentage of coincidence of the top 10 inbred lines list was calculated as described above. For the trait NUpE, 20% and 30% of coincidence between N levels was observed for the multi-trait and single-trait models, respectively, whereas for NUtE, the coincidence was 100% for both models. This result suggests that the trait NUpE was more influenced by the N level than NUtE.

Superior inbred lines in a given environment (N level) for a given trait were identified through the breeding values obtained in the MTME analysis. The superior inbred lines presented greater breeding values for the given trait. The inbred lines that stood out from the others for NUpE were, at the HN level, VML010, VML017, VML077, VML014, VML037, VML009, VML016, VML032, VML005, VML040, whereas at the LN level, VML017, VML051, VML016, VML002, VML033, VML019, VML022, VML188, VML048 and VML034 presented good performances compared to the other inbred lines. For NUtE, the most efficient maize inbred lines were VML081, VML022, L038, VML032, VML051, VML016, VML020, VML027, VML028 and VML013 for both N levels. Another observation that must be reported is that the VML016 inbred line was the only one that appeared in the top 10 list for both traits in the two considered N levels, possibly constituting an important source of genes for NUE traits.

## Conclusions

We demonstrated the feasibility of the proposed multi-trait multi-environment Bayesian model for plant breeding involving a set of genotypes that are evaluated for multiple traits across a range of environments. The accurate estimates of genetic parameters bring new perspectives on the application of Bayesian methods to solve modeling problems in maize breeding.

NUpE was not genetically correlated to NUtE in HN and LN environments. The panel of inbred lines evaluated presents genetic variability, which is fundamental for the selection and improvement of traits of interest. This variability is particularly important in low nitrogen environments, which is the condition that most small farmers that grow maize in the tropics experience. Superior inbred lines were identified for each environment (N level), thus allowing to design selection strategies specifically for each evaluated environment.

## Supporting information

S1 Table Data set necessary to replicate the findings of our research. (TXT)

## References

[1] Ranum P, Pena-Rosas JP, Garcia-Casal MN. Global maize production, utilization, and consumption. Ann NY Acad Sci. 2014; 1312:105–112. doi: 10.1111/nyas.12396 24650320 10.1111/nyas.12396

[2] Pingali PL, Pandey S. World Maize Needs Meeting: Technological Opportunities and Priorities for the Public Sector In: Pingali PL, editor. 1999–2000 World Maize Facts and Trends. Meeting World Maize Needs: Technological oportunities and Priorities for the Public Sector. CIMMYT; 2001 pp 1–24.

[3] Moll RH, Kamprath EJ, Jackson WA. Analysis and interpretation of factors which contribute to efficiency of nitrogen utilization. Agron J. 1982; 74:562–564. doi: 10.2134/agronj1982.00021962007400030037x

[4] Liu J, You L, Amini M, Obersteiner M, Herrero M, Zehnder AJB, et al A high-resolution assessment on global nitrogen flows in cropland. P Natl Acad Sci USA. 2010; 107:8035–8040. doi: 10.1073/pnas.0913658107 10.1073/pnas.0913658107 PMC2867927 20385803

[5] Ciampitti IA, Vyn TJ. Understanding Global and Historical Nutrient Use Efficiencies for Closing Maize Yield Gaps. Agron J. 2014; 106:2107–2117. doi: 10.2134/agronj14.0025

[6] Gallais A, Coque M. Genetic variation and selection for nitrogen use efficiency in maize: a synthesis. Maydica. 2005; 50:531–547.

[7] Presterl T, Seitz G, Landbeck M, Thiemt EM, Schmidt W, Geiger HH. Improving Nitrogen-Use Efficiency in European Maize: Estimation of Quantitative Genetic Parameters. Crop Sci. 2003; 43:1259–1265. doi: 10.2135/cropsci2003.1259

[8] Worku M, Bazinger M, Schulte G, Friesen D, Diallo AO, Horst WJ. Nitrogen uptake and utilization in contrasting nitrogen efficient tropical maize hybrids. Crop Sci. 2007; 47:519–528. doi: 10.2135/cropsci2005.05.0070

[9] Wu Y, Liu W, Li X, Li M, Zhang D, Hao Z, et al Low-nitrogen stress tolerance and nitrogen agronomic efficiency among maize inbreds: comparison of multiple indices and evaluation of genetic variation. Euphytica. 2011; 180:281–290. doi: 10.1007/s10681-011-0409-y

[10] Bazinger M, Betran FJ, Lafitte HR. Efficiency of high-nitrogen selection environments for improving maize for low-nitrogen target environments. Crop Sci. 1997; 37:1103–1109. doi: 10.2135/cropsci1997.0011183X003700040012x

[11] Coque M, Gallais A. Genomic regions involved in response to grain yield selection at high and low nitrogen fertilization in maize. Theor Appl Genet. 2006; 112:1205–1220. doi: 10.1007/s00122-006-0222-5 16552555 10.1007/s00122-006-0222-5

[12] Bertin P, Gallais A. Genetic variation for nitrogen use efficiency in a set of recombinant inbred lines. I. Agrophysiological results. Maydica. 2000; 45:53–66.

[13] Hirel B, Bertin P, Quilleré I, Bourdoncle W, Attacnant C, Dellay C, et al Towards a better understanding of the genetic and physiological basis for nitrogen use efficiency in maize. Plant Phisiol. 2001; 125:1258–1270. doi: 10.1104/pp.125.3.1258 10.1104/pp.125.3.1258 PMC65606 11244107

[14] Moll RH, Kamprath EJ, Jackson WA. Development of nitrogen efficient prolific hybrids of maize. Crop Sci. 1987; 27:181–186. doi: 10.2135/cropsci1987.0011183X002700020007x

[15] Malosetti M, Ribaut JM, Vargas M, Crossa J, Eeuwijk FA van. A multi-trait multi-environment QTL mixed model with application to drought and nitrogen stress trails in maize (*Zea mays* L.). Euphytica. 2008; 161:241–257. doi: 10.1007/s10681-007-9594-0

[16] Hayashi T, Iwata H. A Bayesian method and its variational approximation for prediction of genomic breeding values in multiple traits. BMC Bioinformatics. 2003; 14:34–47. doi: 10.1186/1471-2105-14-34 10.1186/1471-2105-14-34 PMC3574034 23363272

[17] Sorensen DA, Gianola D. Likelihood, Bayesian and MCMC methods in quantitative genetics: statistics for biology and health Springer-Verlag; 2002.

[18] Arriagada O, Mora F, Dellarosa JC, Ferreira MFS, Cervigni GDL, Schuster I. Bayesian mapping of quantitative trait loci (QTL) controlling soybean cyst nematode resistant. Euphytica. 2012; 186:907–917. doi: 10.1007/s10681-012-0696-y

[19] Cané-Retamales C, Mora F, Vargas-Reeve F, Perret S, Contreras-Soto R. Bayesian threshold analysis of breeding values, genetic correlation and heritability of flowering intensity in *Eucalyptus cladocalyx* under arid conditions. Euphytica. 2011; 178:177–183. doi: 10.1007/s10681-010-0292-y

[20] Junqueira VS, Peixoto LA, Galvêas Laviola B, Bhering LL, Mendonça S, Costa TSA, et al Bayesian Multi-Trait Analysis Reveals a Useful Tool to Increase Oil Concentration and to Decrease Toxicity in *Jatropha curcas* L. PLoS One. 2016; 11:1–14. doi: 10.1371/journal.pone.0157038 10.1371/journal.pone.0157038 PMC4900661 27281340

[21] Mora F, Serra N. Bayesian estimation of genetic parameters for growth, stem straightness, and survival in *Eucalyptus globulus* on an Andean Foothill site. Tree Genet Genomes. 2014; 10:711–719. doi: 10.1007/s11295-014-0716-2

[22] Santos MX, Pacheco CAP, Gama EEG, Magnava R, Guimarães PE. Melhoramento da População de milho CMS28. In: Relatório técnico anual do centro nacional de pesquisa de milho e sorgo—1988–1991. Embrapa-CNPMS; 1992 pp 137–138.

[23] Machado AT, Magalhães HR, Magnavaca R, Silva MR. Determinação das atividades de enzimas envolvidas no metabolismo do nitrogênio em diferentes genótipos de milho. Brazilian Journal of Plant Physiology. 1992; 4:45–47.

[24] Elias HT, Carvalho SP, Andre CGM. Comparação de testadores na avaliação de famílias S_2_ de milho. Pesqui Agropecu Bras. 2000; 35:1135–1142.

[25] Naspolini Filho V, Gomes EE, Vianna RT, Môro JR. General and specific combining ability for yield in a diallel cross among 18 maize populations. Braz J Genet. 1981; 4:571–577.

[26] Bremner JM, Mulvaney CS. Nitrogen–Total In: Page AL, Miller RA, Keeney DR, editors. Methods of soil analysis–Part 2. 2nd ed American Society of Agronomy; 1982 pp 595–624.

[27] Van Tassel CP, Van Vleck LD. Multiple-trait Gibbs sampler for animal models: flexible programs for Bayesian and likelihood-based (co)variance component inference. J Anim Sci. 1996; 74:2586–2597. 8923173

[28] Hadfield J. MCMC Methods for Multi-Response Generalized Linear Mixed Models: The MCMCglmm R Package. J Stat Softw. 2010; 33:1–22.20808728

[29] Hadfield JD, Nakagawa S. General quantitative genetic methods for comparative biology: phylogenies, taxonomies and multi-trait models for continuous and categorical characters. J Evolution Biol. 2010; 23:494–508. doi: 10.1111/j.1420-9101.2009.01915.x 10.1111/j.1420-9101.2009.01915.x 20070460

[30] Geweke J. Evaluating the accuracy of sampling-based approaches to the calculation of posterior moments In: Bernardo JM, Berger JO, Dawid AP, Smith AFM, editors. Bayesian Statistics 4. Oxford University Press; 1992 pp 625–631.

[31] Smith BJ. boa: an R package for MCMC output convergence assessment and posterior inference. J Stat Softw. 2007; 21:1–37.

[32] Plummer M, Best N, Cowles K, Vines K. CODA: Convergence diagnosis and output analysis for MCMC. R news. 2006; 6:7–11.

[33] Spiegelhalter DJ, Best NG, Carlin BP, Van der Linde A. Bayesian measures of model complexity and fit. J R Stat Soc. 2002; 64:583–640. doi: 10.1111/1467-9868.00353

[34] Medici LO, Pereira MB, Lea PJ, Azevedo RA. Identification of maize lines with constrasting responses to applied nitrogen. J Plant Nutr. 2005; 28:903–915. doi: 10.1081/PLN-200055586

[35] Han M, Okamoto M, Beatty P H, Rothstein S, Good A G. The Genetics of Nitrogen Use Efficiency in Crop Plants. Annu Rev Genet. 2015; 49:269–289. doi: 10.1146/annurev-genet-112414-055037 26421509 10.1146/annurev-genet-112414-055037

[36] Gallais A, Hirel B. An approach to the genetics of nitrogen use efficiency in maize. J Exp Bot. 2004; 55:295–306. doi: 10.1093/jxb/erh006 14739258 10.1093/jxb/erh006

[37] Piepho HP, Möhring J. Computing Heritability and Selection Response From Unbalanced Plant Breeding Trials. Genetics. 2007; 177:1881–1888. doi: 10.1534/genetics.107.074229 18039886 10.1534/genetics.107.074229 PMC2147938

[38] Viana JMS, DeLima RO, Faria VR, Mundim GB, Resende MDV, Silva FF. Relevance of Pedigree, Historical Data, Dominance, and Data Unbalance for Selection Efficiency. Agron J. 2012; 104:722–728. doi: 10.2134/agronj2011.0358

[39] Thompson R, Meyer K. A review of theoretical aspects in the estimation of breeding values for multi-trait selection. Livest Prod Sci. 1986; 15:299–313. doi: 10.1016/0301-6226(86)90071-0

[40] Azevedo CF, Silva FF, Resende MDV, Peternelli LA, Guimarães SEF, Lopes PS. Quadrados mínimos parciais uni e multivariado aplicados na seleção genômica para características de carcaça em suínos. Cienc Rural. 2013; 43:1642–1649.

[41] Silva FF, Rosa GJM, Guimarães SEF, Lopes PS, de Los Campos G. Three-step Bayesian factor analysis applied to QTL detection in crosses between outbred pig populations. Livest Sci. 2011; 142:210–215. doi: 10.1016/j.livsci.2011.07.012

[42] Viana JMS, Sobreira FM, Resende MDV, Faria VR. Multi-trait BLUP in half-sib selection of annual crops. Plant Breeding. 2010; 129:599–604. doi: 10.1111/j.1439-0523.2009.01745.x

